# mRNA or ChAd0x1 COVID-19 Vaccination of Adolescents Induces Robust Antibody and Cellular Responses With Continued Recognition of Omicron Following mRNA-1273

**DOI:** 10.3389/fimmu.2022.882515

**Published:** 2022-06-02

**Authors:** Alexander C. Dowell, Annabel A. Powell, Chris Davis, Sam Scott, Nicola Logan, Brian J. Willett, Rachel Bruton, Morenike Ayodele, Elizabeth Jinks, Juliet Gunn, Eliska Spalkova, Panagiota Sylla, Samantha M. Nicol, Jianmin Zuo, Georgina Ireland, Ifeanyichukwu Okike, Frances Baawuah, Joanne Beckmann, Shazaad Ahmad, Joanna Garstang, Andrew J. Brent, Bernadette Brent, Marie White, Aedin Collins, Francesca Davis, Ming Lim, Jonathan Cohen, Julia Kenny, Ezra Linley, John Poh, Gayatri Amirthalingam, Kevin Brown, Mary E. Ramsay, Rafaq Azad, John Wright, Dagmar Waiblinger, Paul Moss, Shamez N. Ladhani

**Affiliations:** ^1^ Institute of Immunology & Immunotherapy, College of Medical and Dental Sciences, University of Birmingham, Birmingham, United Kingdom; ^2^ Immunisation and Vaccine Preventable Diseases Division, United Kingdom (UK) Health Security Agency, London, United Kingdom; ^3^ Medical Research Council (MRC)-University of Glasgow Centre for Virus Research, Glasgow, United Kingdom; ^4^ University Hospitals of Derby and Burton National Health Service (NHS) Foundation Trust, Derby, United Kingdom; ^5^ East London National Health Service (NHS) Foundation Trust, London, United Kingdom; ^6^ Manchester University National Health Service (NHS) Foundation Trust, Manchester, United Kingdom; ^7^ Birmingham Community Healthcare National Health Service (NHS) Trust, Aston, United Kingdom; ^8^ Nuffield Department of Medicine, Oxford University Hospitals National Health Service (NHS) Foundation Trust, Oxford, United Kingdom; ^9^ University of Oxford, Oxford, United Kingdom; ^10^ Department of General Paediatrics, Evelina London Children’s Hospital, London, United Kingdom; ^11^ The National Children’s Hospital, Tallaght University Hospital, Dublin, Ireland; ^12^ Children’s Neurosciences, Evelina London Children’s Hospital at Guy’s and St Thomas’ National Health Service (NHS) Foundation Trust, King’s Health Partners Academic Health Science Centre, London, United Kingdom; ^13^ Department Women and Children’s Health, School of Life Course Sciences (SoLCS), King’s College London, London, United Kingdom; ^14^ Department of Paediatric Infectious Diseases and Immunology Evelina London Children’s Hospital, London, United Kingdom; ^15^ United Kingdom (UK) Health Security Agency, Manchester Royal Infirmary, Manchester, United Kingdom; ^16^ Bradford Institute for Health Research, Bradford Teaching Hospitals National Health Service (NHS) Foundation Trust, Bradford, United Kingdom; ^17^ Paediatric Infectious Diseases Research Group, St. George’s University of London, London, United Kingdom

**Keywords:** COVID-19, vaccine, paediatric, T-cell, antibody, neuro-disabilities, high-risk patients

## Abstract

Children and adolescents generally experience mild COVID-19. However, those with underlying physical health conditions are at a significantly increased risk of severe disease. Here, we present a comprehensive analysis of antibody and cellular responses in adolescents with severe neuro-disabilities who received COVID-19 vaccination with either ChAdOx1 (n=6) or an mRNA vaccine (mRNA-1273, n=8, BNT162b2, n=1). Strong immune responses were observed after vaccination and antibody levels and neutralisation titres were both higher after two doses. Both measures were also higher after mRNA vaccination and were further enhanced by prior natural infection where one vaccine dose was sufficient to generate peak antibody response. Robust T-cell responses were generated after dual vaccination and were also higher following mRNA vaccination. Early T-cells were characterised by a dominant effector-memory CD4+ T-cell population with a type-1 cytokine signature with additional production of IL-10. Antibody levels were well-maintained for at least 3 months after vaccination and 3 of 4 donors showed measurable neutralisation titres against the Omicron variant. T-cell responses also remained robust, with generation of a central/stem cell memory pool and showed strong reactivity against Omicron spike. These data demonstrate that COVID-19 vaccines display strong immunogenicity in adolescents and that dual vaccination, or single vaccination following prior infection, generate higher immune responses than seen after natural infection and develop activity against Omicron. Initial evidence suggests that mRNA vaccination elicits stronger immune responses than adenoviral delivery, although the latter is also higher than seen in adult populations. COVID-19 vaccines are therefore highly immunogenic in high-risk adolescents and dual vaccination might be able to provide relative protection against the Omicron variant that is currently globally dominant.

## Introduction

SARS-CoV-2 infection in children and adolescents is generally mild, transient and self-limiting, however those with underlying co-morbidities have a higher risk of developing severe and fatal COVID-19 (1–3). As such, vaccination of high-risk children and adolescents against COVID-19 is of considerable importance. Recent studies have indicated mRNA vaccination of adolescents is highly protective against severe and critical COVID-19 (4–6), including in high risk groups (7). The immunogenicity of COVID-19 vaccines in high-risk paediatric groups, however, has not been assessed.

COVID-19 vaccines were first licensed for adults in December 2020 and have proven to be highly effective. In the United Kingdom, the Joint Committee on Vaccination and Immunisation (JCVI) recommended COVID-19 vaccination for older children aged ≥12 years with severe neuro-disabilities at the same time as adults, even though the vaccines were not authorised for this age-group at the time, because they were at higher risk of severe and fatal COVID-19 (1). In March 2021, Public Health England (PHE) (now known as the UK Health Security Agency) initiated the SAFE-KIDS study to assess immune responses in children receiving a COVID-19 vaccine as part of the JCVI recommendation. At the time Moderna (mRNA-1273), and Pfizer-BioNTech (BNT162b2) mRNA vaccines and the AstraZeneca adenoviral-vector (ChAdOx1) vaccine were recommended for adults and high-risk adolescents. Contrary to the marketing authorisation of 3-4 weeks, the JCVI recommended a 12-week interval between COVID-19 vaccine doses. As such, this provided a unique opportunity to compare after one and two doses the relative immunogenicity of adenoviral vector vaccines to mRNA vaccines in adolescence. Currently no comparative data exist.

**Figure 1:**
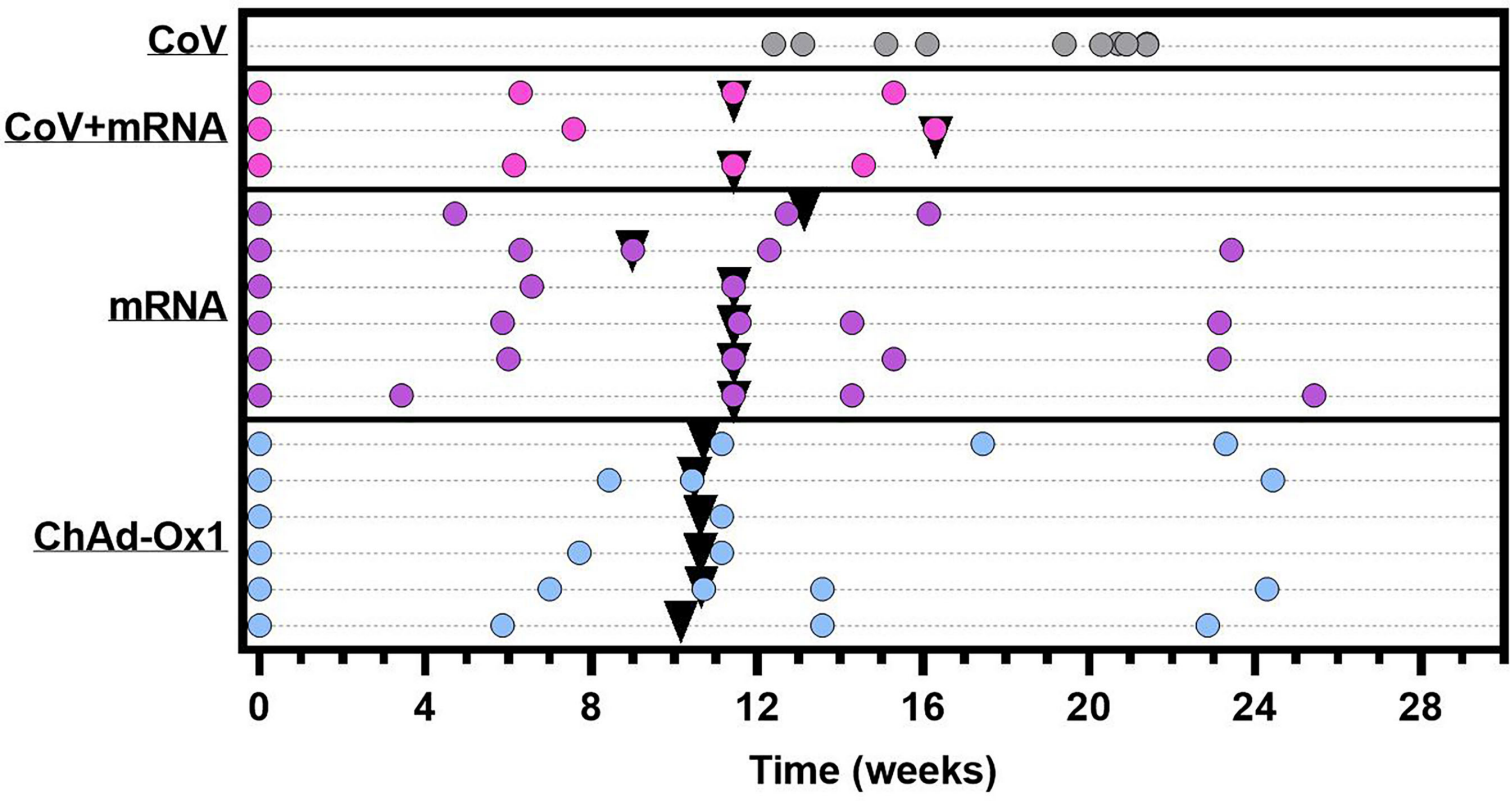
Graphical representation of sample collection and vaccine administration. CoV+mRNA (pink dots) = seropositive adolescents receiving mRNA vaccination (n = 3) mRNA (purple dots) = seronegative adolescents receiving mRNA vaccination (n = 6). ChAdOx1 (blue dots) = seronegative adolescents receiving ChAdOx1 vaccination (n = 6). CoV (grey dots) = naturally infected adolescents with definitive PCR results, for comparison (n = 10). Each dot represents a sample collection. Black triangles indicate time of second dose. Time for all vaccinated donors is relative to administration of first dose. Time of blood sampling for naturally infected donors (CoV) is relative to the date of PCR.

Here, we provide detailed characterisation of the antibody and cellular immune response to COVID-19 vaccination in fifteen adolescent individuals with severe neuro-disabilities aged 12-16 years. Uniquely, donors received either ChAdOx1 (n=6) or mRNA vaccine (n=8 mRNA-1273, n=1 BNT162b2). Three donors receiving mRNA-1273 had serological evidence of SARS-CoV-2 infection prior to vaccination. Participant characteristics are summarised in **Supplementary Table 1**, with details of vaccination and sample timing in **Figure 1**.

## Results

### Characterization of Antibody Titres Following First and Second Dose of COVID-19 Vaccination

**Figure 2:**
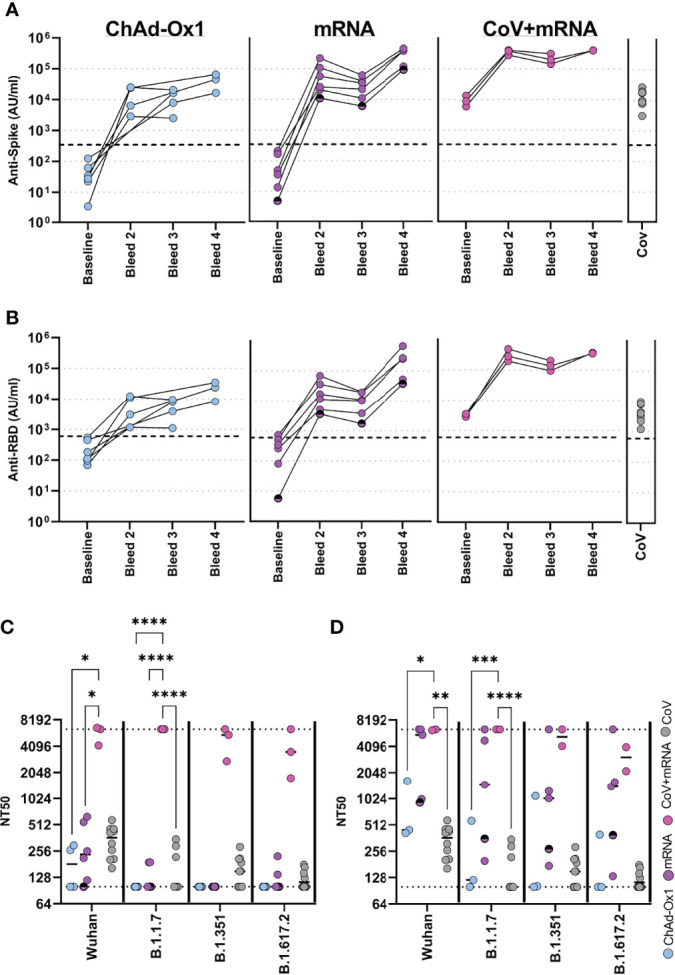
Antibody responses in adolescents following COVID-19 vaccination. Antibody levels to Spike **(A)** and RBD **(B)** measured by MSD assay in adolescents receiving COVID-19 vaccination (ChAdOx1 – seronegative adolescents receiving ChAdOx1 vaccination, (n = 6), mRNA – seronegative adolescents receiving mRNA vaccination (n = 6), CoV+mRNA – seropositive adolescents receiving mRNA vaccination (n = 3), half shaded mRNA symbol indicates the individual who received BNT162b2 vaccine). Antibody levels 2-4 months after natural SARS-CoV-2 infection [CoV, (n = 10)] are shown for comparison. **(C**, **D)** Neutralisation of live virus, either B (Wild Type; PHE-2), B.1.1.7 (Alpha), B.1.351 (Beta) or B.1.617.2 (Delta) variants, following first dose **(C)** or second dose **(D)**. Dotted lines represent upper and lower limits of detection. RM Two-way ANOVA with Geisser-Greenhouse correction and Tukey’s multiple comparison test. *p<0.05, **p<0.01, ***p<0.001, ****p<0.0001.

We firstly determined the antibody response using the Meso-Scale Diagnostics (MSD) assay platform, allowing comparison with other studies using the same platform (8, 9). Samples were assessed longitudinally from baseline. All vaccines induced robust spike-specific (**Figure 2A**) and receptor-binding domain- (RBD-) specific (**Figure 2B**) antibodies. mRNA vaccine, however, induced 4.4-fold and 6.6-fold higher spike-specific antibody responses than the adenovirus-based vaccine after the first and second dose, respectively. Individuals who were previously naturally infected prior to vaccination demonstrated a marked increase in antibody levels after the first mRNA vaccine. Spike-specific antibody levels were significantly higher than one or two doses of ChAdOx1, and significantly higher than one dose of mRNA vaccine (p=0.001, 0.004 and 0.003 respectively, one-way ANOVA with Dunnett’s multiple comparisons test). Two doses of mRNA vaccine in previously uninfected individuals achieved similar antibody levels to donors who had received one dose of vaccine after prior natural infection. Antibody levels for all cohorts are shown relative to the WHO reference standard in **Supplementary Table 2**. Additionally, antibody titres specific for the seasonal human coronaviruses, Influenza-A, -B, and Respiratory Syncytial Virus (common respiratory viruses), were determined and found to be comparable to healthy donors (**Supplementary Table 3**).

We next assessed functional neutralisation using live wild-type (B), Alpha (B1.1.7), Beta (B1.351) or Delta (B1.617.2) viral variants. For comparison, we also included samples from 10 healthy adolescent donors who had PCR-confirmed SARS-CoV-2 infection 2-4 months before sampling. After the first dose of vaccine, in naïve donors, neutralisation titres (NT50) were similar or below that of natural infection against WT virus. Neutralisation of variants was in general below detection. In contrast, previously infected donors induced high neutralising titres to wild type and all viral variants after one dose (**Figure 2C**). Following second dose in naïve donors, neutralizing titres were improved, including to variants (**Figure 2D**). Comparable results were also seen against these and other variants in a ACE2-Spike binding inhibition assay (**Supplementary Figure 1**). There was considerable variability between donors receiving ChAdOx1 or mRNA vaccine, with the latter inducing higher neutralising titres than the former in this limited cohort.

### Characterization of the Cellular Response Following COVID-19 Vaccination

**Figure 3:**
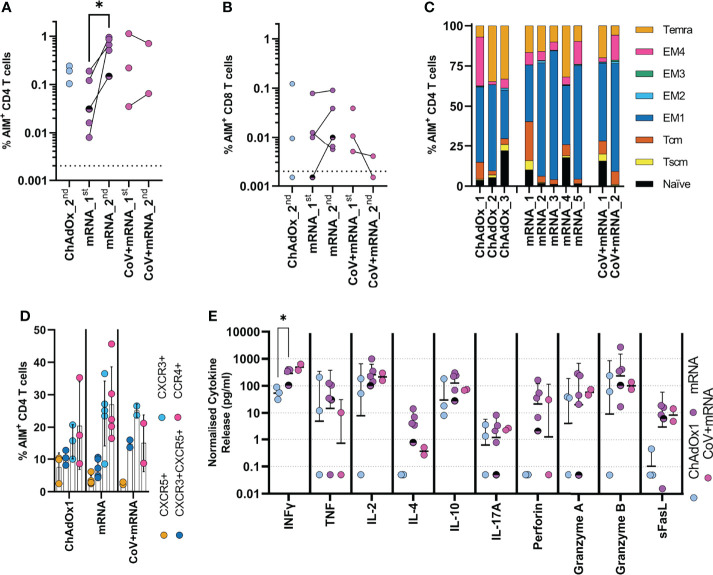
Initial cellular responses in adolescents following COVID-19 vaccination. Analysis of the T-cell response following the second dose of vaccine. A flow cytometry-based AIM assay was used to identify responding **(A)** CD4 (CD69+CD40L+) and **(B)** CD8 (CD69+CD137+) T-cell frequency following overnight stimulation with an overlapping spike peptide pool. Where possible frequency was assessed 6 weeks after first dose _1^st^ or 3 weeks after second dose _2^nd^. Dots indicate individual donors. n = 3 ChAdOx1 (ChAdOx), n = 5 mRNA and n = 2 CoV+mRNA, half shaded mRNA symbol indicates the individual who received BNT192b2 vaccine. **(C)** Responding AIM+ CD4 T-cells from donors after second dose, were phenotyped, to assess memory state. Bars represent individual donors receiving the indicated vaccine type, numbers indicate individual donors and show the proportion of the AIM+ CD4 T-cell population with each memory phenotype in each donor. T effector memory (CD45RA-CCR7-, EM) are subdivided as EM1 – CD27+CD28+, EM2 – CD27+CD28-, EM3 – CD27-CD28-, EM4 - CD27-CD28+). **(D)** the expression of homing and polarisation markers CXCR5, CXCR3, and CCR4 by AIM+ CD4 T-cell population was also assessed and expressed as a proportion of the total AIM+ CD4 T-cell population. Dots indicate individual donors, bars indicate mean ± SD. **(E)** Supernatant from overnight stimulated cultures were analysed to identify cytokine production. Data was normalised to 1x10^6^ PBMC per well and minus background cytokine production from unstimulated (DMSO) wells. Dots indicate individual donors; bars indicate geometric mean ± geo.SD. Repeated measure two-way ANOVA with Geisser-Greenhouse correction and Tukey multiple comparisons test. *p<0.05.

We next investigated the cellular immune response after two vaccine doses (median 23-days, range 20-48), in three ChAdOx1 and seven mRNA vaccinated adolescents, including two with prior infection. The timing of sampling is shown in **Figure 1**. Spike-specific cellular responses were assessed using an activation-induced marker (AIM) assay to identify T-cells responding to stimulation with a pool of overlapping-peptides from spike protein, an example is provided in **Supplementary Figure 2**. As previously reported in adults (10), responses were dominated by CD4 T-cells, with ~10-fold lower CD8 T-cell responses. The CD4 T-cell response was higher in mRNA-1273 vaccinated adolescents compared to those vaccinated with ChAdOx1 (**Figures 3A, B**). Cellular samples were also available after one vaccine dose for adolescents receiving mRNA vaccine, allowing the trajectory of T-cell responses to be assessed. The T-cell response appeared to peak after one dose in the two previously-infected donors. In contrast, the CD4 T-cell response increased significantly (p=0.047, two-tailed paired t-test) following second dose in infection-naïve donors (**Figure 3A**). Characterisation of the CD4 T-cell response in more detail showed, in all vaccine types, the predominant phenotype was early (CD27+CD28+) effector memory, with elements of central memory, indicating induction of a memory response (**Figure 3C**). CXCR3 and CCR4 expression characterise Th1 and Th2 CD4+ populations, respectively, and both were represented within the spike-specific response at similar frequencies. CXCR5+ T follicular helper cells were also present within the antigen-specific pool (**Figure 3D**).

The cytokine profile of spike-specific T-cells was determined by analysis of supernatant from AIM assay cultures. Type-I cytokines were found at high levels, with IFNγ and IL-2 predominating; release of TNF was also notable, consistent with a Type-I cytokine profile. The level of IFNγ was significantly higher in mRNA vaccinated donors compared to ChAdOx1 (p=0.016, RM two-way ANOVA with Geisser-Greenhouse correction and Tukey’s multiple comparison test), consistent with the higher frequency of responding cells. Cells also demonstrated cytotoxic potential, with release of perforin, granzyme A and B. In contrast, high IL-10 concentrations were also found within the supernatant. There was, however, little evidence of IL-4 production, with levels generally below 10pg/ml, although it is interesting to note increased detection of IL-4 in mRNA-vaccinated individuals which may be associated with the increased proportion of responding cells. This level of IL-4 is not consistent with the high proportion of activated CCR4-expressing cells being Th2-polarised. IL-17A was also detected but below 10pg/ml (**Figure 3E**). The profile of cytokine release did not change between first and second dose in mRNA vaccinated children (**Supplementary Figure 3**) and was consistent between naïve and previously-infected individuals.

### Durability of Antibody Responses and Neutralisation of Omicron Variant 3 Months Post-Vaccination

**Figure 4:**
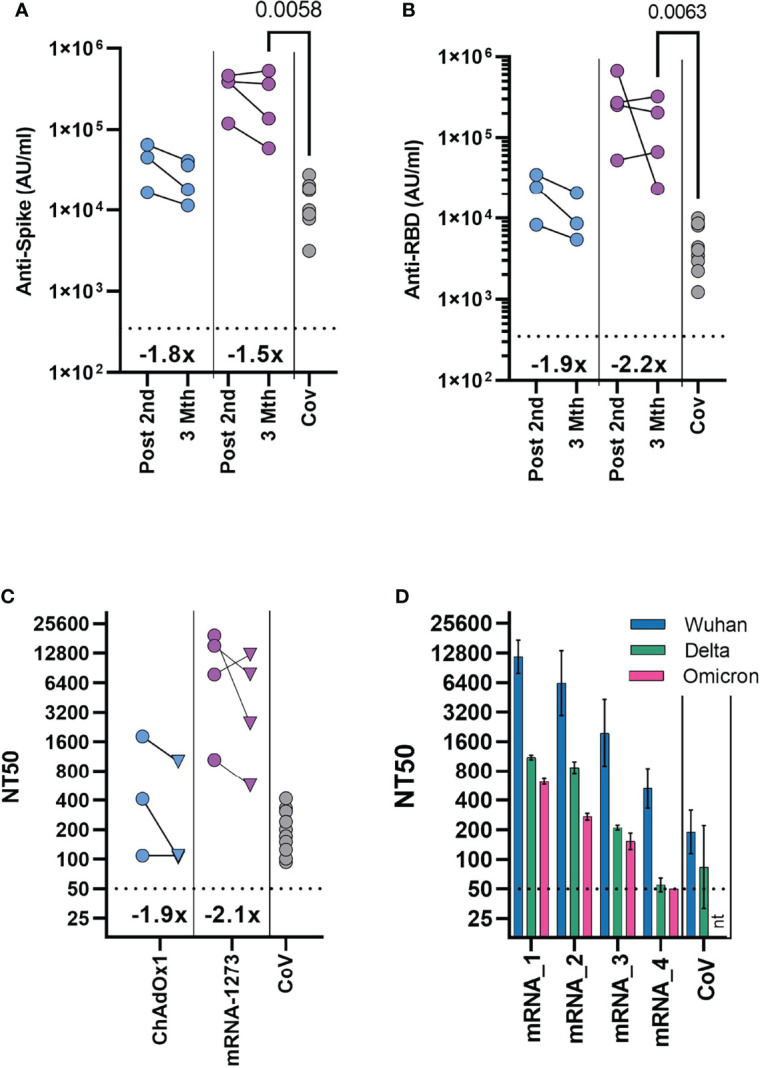
Durability of antibody response and neutralisation of the Omicron variant 3 months after second dose. Antibody levels to Spike **(A)** and RBD **(B)** measured by MSD assay in previously seronegative adolescents receiving two COVID-19 vaccinations (ChAdOx1 – (n = 3), mRNA-1273 – (n = 4), either following second dose (Post 2^nd^), or three months after second dose (3 Mth). Antibody levels 2-4 months following natural SARS-CoV-2 infection [CoV, (n = 10)] are shown for comparison. **(C)** Neutralising antibody titres quantified using HIV (SARS-CoV-2) pseudotypes bearing the Wuhan spike glycoprotein. Each point represents the mean of three replicates, circles indicate following second dose, triangle three months after second dose. **(A–C)** Inset show the fold change in geometric mean titre. **(D)** Neutralising antibody titres quantified three months after second dose in four mRNA-1273 vaccinated individuals using HIV (SARS-CoV-2) pseudotypes bearing either Wuhan, B.1.617.2 (Delta) or B.1.1.529 (Omicron) spike glycoprotein. mRNA1-4 indicate individual donors. Bars indicate mean (± geometric SD). Neutralisation titres 2-4 months following natural SARS-CoV-2 infection [CoV, (n=10)] are shown for comparison. Kruskal-Wallis test with Dunn’s multiple comparison correction.

Rapid waning of the spike-specific antibody level following vaccination is evident in adults, with the greatest decline seen in the initial period following mRNA vaccination (11, 12). In vaccinated adolescents, there was also reduction of the spike-specific antibody level between first and second dose of mRNA vaccination of naïve adolescent donors (**Figure 1A**). Irrespective of vaccine type, modest reduction of the spike-specific antibody level was evident 3 months after the second dose, with a 1.5- and 1.8-fold decrease in mRNA and ChAdOx1 vaccinated adolescents respectively (**Figure 4A**). A greater reduction was evident in RBD-specific antibody levels with a 2.2 - and 1.9-fold reduction following mRNA and ChAdOx1 vaccination respectively (**Figure 4B**).

A direct comparison could now be made between vaccinated donors and the cohort of naturally infected adolescents, as these donors were at a similar timepoint after infection. Antibody levels were significantly higher in the mRNA-1273 vaccinated individuals, compared to naturally infected individuals (p=0.0058, Kruskal-Wallis test with Dunn’s multiple comparisons test). Spike-specific antibody levels were 2.1 and 18-fold higher than the naturally infected donors in the ChAdOX1 and mRNA-1273 vaccinated groups, respectively (**Figure 4A**; **Supplementary Table 2**).

We tested whether neutralising antibody titres were retained to a similar degree as total spike- and RBD-specific antibodies at three months after the second dose, using a pseudotyped virus neutralisation assay. Reduction of neutralising titres was evident in both ChAdOx1 and mRNA-1273 vaccinated individuals, compared to titres after the second vaccine dose (**Figure 4C**). Neutralising titres were reduced by 2.1- and 1.9-fold in mRNA and ChAdOx1 vaccinated adolescents respectively, comparable to the reduction in RBD-specific antibody levels. Neutralisation titres from mRNA-1273 vaccinated individuals, however, remained significantly higher than titres in healthy adolescents 2-4mths after natural infection (n=10), (p=0.019, Kruskal-Wallis test with Dunn’s multiple comparisons test), although considerable variability in titre was evident. Titres from ChAdOx1 vaccinated donors were also similar to natural infection.

The emergence of the Omicron (B.1.1.529) variant has reduced the effectiveness of vaccine protection against infection after two doses of vaccine in adults, requiring further booster vaccinations to elicit higher antibody titres (13, 14). As such, understanding of the longer-term neutralisation of Omicron in vaccinated adolescents is vital. Given this, we tested the neutralisation of Omicron variant in mRNA-1273 vaccinated individuals three months after vaccination. Titres were reduced against Omicron in comparison to the Wuhan sequence, with considerable variation between donors although, importantly, 3 of 4 individuals had measurable neutralisation titres against Omicron (**Figure 4D**). Indeed, titres from individuals with measurable neutralisation titres against Omicron, were similar to or above titres against Wuhan spike from naturally infected healthy adolescents.

### Durability of the T-Cell Response and Response to the Omicron Variant 3 Months After Second Dose

**Figure 5:**
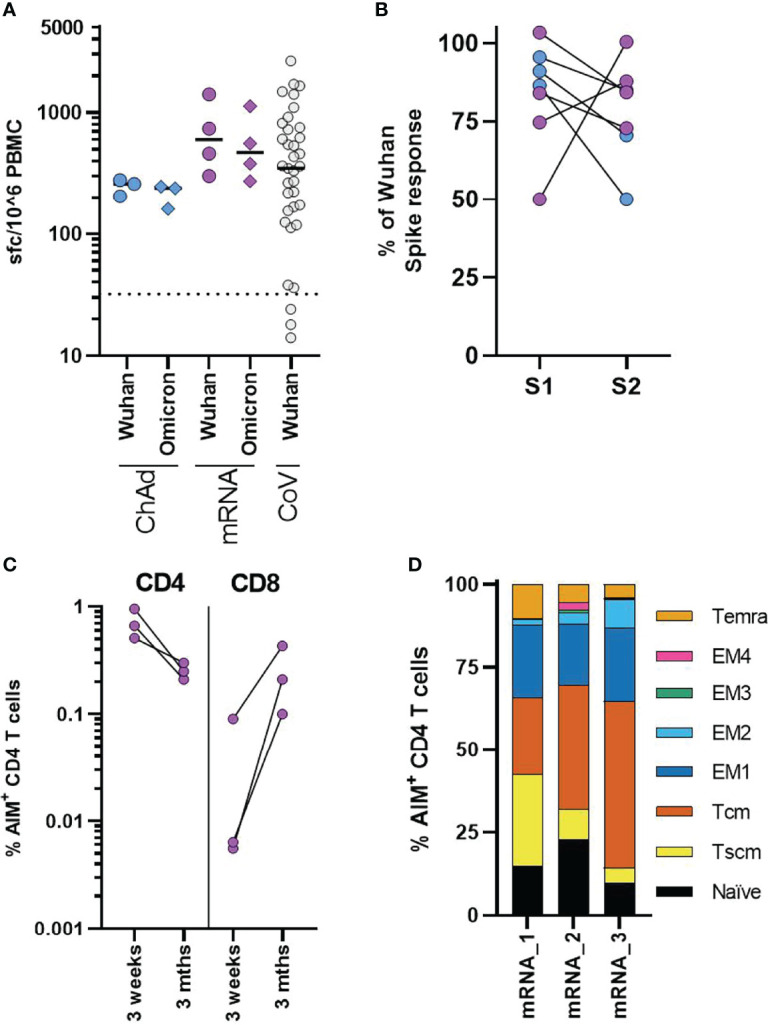
Cellular responses in adolescents three months after COVID-19 vaccination. **(A)** Analysis of the T-cell response in ChAdOx1 (n = 3) and mRNA-1273 (n = 4) adolescents three months after second dose. Results from IFNγ ELISpot assay are normalised to the DMSO control and expressed as spot forming cells (sfc) per million input PBMC. Response to overlapping peptide pools from Wuhan (circles) or B1.1.529 (diamonds) sequence spike protein. Results from previously published data from n = 37 seropositive children aged 4-11 are shown for comparison, dotted line indicates response threshold. **(B)** Shows the relative change in the response to peptide pools containing peptides from the S1 and S2 spike protein domains of Omicron, expressed relative to the size of the response to Wuhan sequence peptide pools. **(C**, **D)** A flow cytometry-based AIM assay was used to identify responding CD4 (CD69+CD40L+) and CD8 (CD69+CD137+) T-cell frequency following overnight stimulation with overlapping Wuhan spike peptides in three adolescents who received mRNA-1273 vaccination. **(C)** Dots indicate individual donors and frequency of AIM+ CD4 and CD8 T-cells following second dose of vaccine (3 weeks) or three months after second dose (3 Mths). **(D)** AIM+ CD4 T-cells, were phenotyped to assess memory state, bars represent indicate individual donors (numbered 1-3) and show the proportion of the AIM+ CD4 T-cell population. T effector memory (CD45RA-CCR7-, EM) are subdivided as EM1 – CD27+CD28+, EM2 – CD27+CD28-, EM3 – CD27-CD28-, EM4 - CD27-CD28+).

Finally, we examined the T-cell response in three ChAdOx1 and four mRNA-1273 adolescents who had cellular samples available for assessment three months after second vaccination. We assessed responses to pools of overlapping peptides from either Wuhan or Omicron (B.1.1.529) sequence spike protein using IFNγ ELISpot. Consistent with the initial response measured by AIM assay, T-cell responses were higher in mRNA-1273 vaccinated donors, although variation was evident between donors. Importantly all donors irrespective of vaccine type retained robust T-cell responses to Wuhan peptide pools 3 months after vaccination, similar to that previously observed in children following natural infection (**Figure 5A**) (15). In comparison to the Wuhan sequence, the T-cell response was reduced to Omicron spike peptide pool, with the response on average 84% (range 92.2-76%) of the response to the Wuhan spike peptide pool (**Figure 5A**). The majority of mutations in the Omicron spike sequence occur in the S1 domain of the spike protein (14). Comparing the response to peptides derived from the S1 and S2 domains of Wuhan and Omicron (B.1.1.529) spike sequence, we found that, while the reduction in total spike response was relatively consistent across donors, the relative reduction in response to S1 and S2 domains showed a degree of donor variation (**Figure 5B**).

We again used the AIM assay to assess the phenotype of T-cell response at three months. Sufficient cellular samples were available from three mRNA-1273 vaccinated donors. These donors were also assessed ~3 weeks after the second vaccine dose by AIM assay (**Figure 3A–C**). The CD4 T-cell response was found to be lower in all donors, compared with the earlier peak response. In contrast, an increase in the frequency of AIM+ CD8 T-cells was observed (**Figure 5C**). Initially following vaccination, AIM+ CD4 T-cells were predominantly effector memory (CD45RA-CCR7-) phenotype (**Figure 3C**) but, three months after vaccination AIM+ CD4 T-cells were predominantly central (CD45RA-CCR7+) and stem cell memory (CD45RA+CCR7+CD95+) phenotype in all donors (**Figure 5D**). There was a corresponding reduction in T effector/effector memory phenotype cells. These data, therefore, indicate the successful formation of long-term memory T-cell responses following vaccination.

## Discussion

The UK JCVI decision to recommend COVID-19 vaccination for adolescents with neuro-disabilities, soon after COVID-19 vaccines were authorised for adults, provided a unique opportunity to rapidly assess immune responses to two different types of vaccines in this high-risk group in a real-world setting. While there is growing evidence of clinical efficacy of vaccination, there are limited data characterising the immune responses to COVID-19 vaccination in children and adolescents, especially in high-risk groups, and none comparing responses in adolescents who received ChAdOx1 against an mRNA vaccine (4–7). Uniquely, instead of the 3-4 week interval used by most other countries, the JCVI recommendations included a 12-week interval between COVID-19 vaccine doses, to maximise population first dose coverage. In adults, it is now evident that an extended interval schedule provides higher peak antibody responses after the second dose and, potentially, longer duration of protection (16). Importantly, the extended interval also provided a unique opportunity to assess the immunogenicity of the initial vaccine dose, prior to the second dose.

The higher immunogenicity with mRNA vaccine we observed compared to ChAdOx1 vaccine is consistent with data in adults (8, 9, 11, 17), including studies reporting the extended 12-week interval schedule (8, 16). The antibody level after mRNA vaccination was comparable to those previously reported in adults (8), but higher in adolescents than in adults after ChAdOx1 vaccination (9). It is noted the single donor who received BNT162b2 had similar antibody levels as ChAdOx1 vaccinated donors. In adults, the highest antibody levels are reported following mRNA-1273 followed closely by BNT162b2 (13). Further study will be required to define the relative antibody response induced by mRNA-1273, BNT162b2, and ChAdOx1 in larger adolescent cohorts. Our preliminary data, however, indicate that ChAdOx1 may be more immunogenic in adolescents than adults, and confirm that both vaccine types provide robust antibody responses using an extended-interval schedule in these higher-risk adolescents.

Rapid waning of spike-specific antibody level following vaccination is evident in adults, with the greatest decline seen following mRNA vaccination, and the most rapid waning occurring soon after vaccination (11, 12). It was, therefore, encouraging to observe comparatively stable antibody responses between 21 days (range 20-27 days) after the second dose and three months later. Antibody titres reduced by 1.5-fold, compared to an approximate 3.7-fold decline reported in adults receiving an extended-interval BNT162b2 vaccine schedule, measured at similar timepoints using the MSD platform (8). These data showing retained antibody titres are consistent with the better clinical protection in adolescents 3 months after vaccination compared to adults (6).

A key aim of vaccination is the induction of neutralising antibodies. The use of a 12-week vaccination schedule uniquely allowed us to explore the relative immunogenicity of one and two vaccine doses in adolescents. Our data show that a second dose significantly increases immune responses in infection-naïve adolescents, with improved neutralising titres after the second vaccine dose, including to the viral variants tested. While mRNA vaccines induced higher neutralising titres, there was considerable variability between donors, again with the BNT162b2 vaccinated donor being similar to ChAdOx1. We also assessed neutralisation at three months after vaccination, an important point at which to evaluate the durability of response, given the waning in protection observed in adults (11, 18). We chose to use a pseudotype neutralisation assay as isolates of Omicron were not yet available. Both live virus and pseudotype assays give comparable results and are regarded to be well correlated (19), as such these results should be representative of live virus neutralisation assays. Despite the relatively stable total spike-specific antibody responses, there was evidence of waning of neutralisation titres, which mirrored the reduction in RBD-specific-antibody level highlighting the need for further long-term assessment in larger groups of adolescents and children. We have previously shown that antibody responses in children are durable up to 12 months after natural infection (15).

Given the ability of Omicron to evade vaccine immunity (13, 14), it was vital to assess the immune response against this variant. Three months after vaccination provided an informative point to address this. As in adults, Omicron-neutralisation titres were significantly reduced compared to Wuhan (13, 14). However, titres were similar to those seen in adults after booster mRNA vaccination (13), indicating comparable neutralising response in dual vaccinated adolescents. Surprisingly, mRNA-1273 vaccinated adolescents who retained a measurable Omicron neutralising titre, had similar or enhanced titres compared to neutralising titres against Wuhan spike in naturally infected healthy adolescents, highlighting that mRNA-1273 vaccination of adolescents, and likely children, may offer improved and broader immunity to Omicron than natural infection. However, further evaluation of neutralisation titres in larger cohorts and real-world epidemiological studies are required.

Cellular immune responses are ultimately likely to be more durable than antibody responses and provide longer-term protection against severe disease (20, 21). Additionally, T-cells are likely to be less susceptible to changes in viral variants (22). Importantly, we found that, while T-cell responses were reduced towards the Omicron spike sequence when compared to the neutralising antibody response, the T-cell response was retained at a robust level towards the Omicron variant spike peptide pool. The relative reduction in responses to S1 and S2 domains showed donor variation, potentially indicating differences in the epitope and immunodominance of epitopes between donors. The T-cell response was observed to peak after one dose in the two previously-infected children, consistent with results from adults (23), although a larger cohort is required to fully assess this. The CD4 T-cell response in infection-naïve adolescents was, however, significantly enhanced by a second dose of vaccine, again indicating the requirement for two vaccine doses in infection-naïve adolescents.

The T-cell response retained at three months after vaccination was similar to the range of responses observed after SARS-CoV-2 infection in younger children aged 4-11 years, which was also significantly higher than in adults (15). As such, this showed that vaccination elicits robust T-cell responses in adolescents similar to that produced following natural infection. Given the range of responses following natural infection, larger cohorts will be required to assess whether the lower response after ChAdOx1 is a result of differences between vaccine type or donor variation. However, the finding that the mRNA-1273 vaccine generates strong spike-specific cellular responses in adolescents contrasts with previous reports in older adults where cellular responses after ChAdOx1 are somewhat higher than after mRNA vaccination (8, 9, 24). Cellular responses displayed an effector memory phenotype early after the second vaccine, representing recent antigenic stimulation (25), and it was therefore reassuring to observe the formation of a potential long-term memory pool three months later.

Spike-specific T-cells displayed a predominant Type-I cytokine profile, but high IL-10 concentrations were also observed and is noteworthy as this combination has been reported previously as a feature of mild or asymptomatic infection (10, 15, 26). The presence of spike-specific T-cells expressing CCR4 in the absence of IL-4 may also be important as this is a specific phenotypic profile of lung/skin homing (27) rather than Th2 polarisation.

A limitation of this study is the small number of participants, mainly due to very low vaccine uptake in eligible groups during the first 6 months of 2021 (28). While considered as high-risk because of severe neuro-disabilities, our participants are clinically immune competent and their antibody levels against other respiratory viruses were comparable to healthy donors, indicating normal immune function. As such the robust immune responses to SARS-CoV-2 vaccine in these donors should be representative of the general adolescent population. One participant did have Down syndrome, which is associated with unspecified immune dysfunction (29), although our participant mounted a robust immune response after vaccination.

Our study provides a detailed and unique insight into the relative immunogenicity of mRNA and adenoviral-vector vaccines in infection-naïve and previously infected adolescents, following one and two doses of vaccine. Overall, these findings show that COVID-19 vaccination in adolescents can elicit coordinated and durable cellular and antibody responses with activity against Omicron variant. Further investigation in larger cohorts is required to confirm these findings.

## Methods

### Sample Collection

Eligible children (**Supplementary Table 1**) aged 12-16 years were recruited into SAFE-KIDS by paediatricians. The current study was reviewed and approved by the PHE Research Ethics and Governance Group (REGG) reference NR0264. Written informed consent was obtained for all participants from parents or guardians.

Blood samples taken at baseline (before or within 48 hours of first vaccination), ~6 weeks follow prime (bleed 2), at boost (bleed 3), ~2-4 weeks following boost (bleed 4) and ~3 months following boost (bleed 5) (**Figure 1**). The vaccine administered was not pre-determined, children received vaccine as part of the national vaccine campaign, as such vaccine type was dependent on availability at each site. The full SAFE-KIDS protocol is available online at https://www.gov.uk/guidance/covid-19-paediatric-surveillance.

Sero-status was determined as described below using the baseline sample. Nucleocapsid-specific antibody responses were assessed at each timepoint to exclude the possibility of SARS-CoV-2 infection during the study. Routine surveillance PCR testing was not performed during this study.

Convalescent plasma samples were also available from the Born in Bradford study (30). 10 children aged 10-13 years with PCR-confirmed SARS-CoV-2 infection between October and December 2020, had samples taken a median of 18 weeks after PCR (**Supplementary Table 1**).

### PBMC and Plasma Preparation

Lithium Heparin blood tubes were processed within 24hrs of collection. Briefly tubes were spun at 300g for 10mins prior to removal of plasma which was then spun at 800g for 10mins and stored as aliquots at -80°C. Remaining blood was diluted with RPMI and PBMC isolated on ficol density gradient, washed with RPMI and frozen in 90%FBS+10%DMSO, samples were stored in the vapour phase of liquid nitrogen.

### Serological Analysis of SARS-CoV-2-Specific Immune Response

Quantitative IgG antibody titres were measured using Mesoscale Diagnostics multiplex assays as previously described (15), following the manufacturer instructions. Briefly, samples were diluted at 1:5000 and added wells of the 96 well plate alongside reference standards and controls. After incubation, plates were washed and anti-IgG-Sulfo tagged detection antibody added. Plates were washed and were immediately read using a MESO TM QuickPlex SQ 120 system. Data was generated by Methodological Mind software and analysed with MSD Discovery Workbench (v4.0) software. Data are presented as arbitrary units (AU)/ml relative to the standard. Anti-Spike and anti-RBD AU/ml were converted to WHO reference standard Binding Antibody units (BAU)/ml using the provided correction values, using the following formula: anti-spike - AU/ml*0.00901=BAU/ml, anti-RBD - AU/ml*0.0272=BAU/ml. Positive and negative cut-off values were used as previously defined (15), using plasma samples taken prior to the pandemic.

### Live Virus Neutralisation Assay

Clinical isolates used in the study were provided by Public Health England and Imperial College London. A549-ACE2-TMPRSS2 (31) cells were seeded at a cell density of 1x10^4^/well in 96-well plates 24hrs before inoculation. Serum was titrated starting at a 1:100 dilution-1:6400 dilution. The specified virus was then incubated at an MOI 0.01 with the Serum for 1hr prior to infection. All wells were performed in triplicate. 72hrs later infection plates were fixed with 8% formaldehyde and stained with Coomassie blue for 30 mins. Plates were washed and dried overnight before quantification using a Celigo Imaging Cytometer (Nexcelom) to measure the staining intensity. Percentage cell survival was assessed by comparing the intensity of the staining to uninfected wells. Antibody titre was then estimated by interpolating the point at which infectivity had been reduced to 50% of the value for the no serum control samples.

### Pseudotype-Based Neutralisation Assays

Constructs and 293-ACE2 cells were previously described (13, 15). The assay was performed as previously described (13, 15), briefly neutralising activity in each sample was measured by a serial dilution approach. Each sample was serially diluted in triplicate from 1:50 to 1:36450 in complete DMEM prior to incubation with approximately 1x10^6^ CPS (counts per second) per well of HIV (SARS-CoV-2) pseudotypes, incubated for 1 hour, and plated onto 239-ACE2 target cells. After 48-72 hours, luciferase activity was quantified by the addition of Steadylite Plus chemiluminescence substrate and analysis on a Perkin Elmer EnSight multimode plate reader (Perkin Elmer, Beaconsfield, UK). Antibody titre was then estimated by interpolating the point at which infectivity had been reduced to 50% of the value for the no serum control samples.

### Spike-ACE2 Receptor Blocking Assay

Inhibition of ACE-2 binding to trimeric SARS-CoV-2 Spike protein from variants of concern were measured using the MSD V-PLEX COVID-19 ACE2 Neutralization Kit (SARS-CoV-2 Plate 13) following manufacturer’s instructions. Briefly, samples were diluted 1:10 in diluent, known neutralizing antibody dilutions were included as a reference standard, and pre-incubated on the plate, which was coated with trimeric spike from SARS-CoV-2 variants. After incubation, Sulfo-tagged Human ACE-2 Protein was added to the plate and incubated for 1 hour. Plates were washed and read immediately using a MESO ™ QuickPlex SQ 120 system. Data was generated by Methodological Mind software and analysed with MSD Discovery Workbench (v4.0) software. Presented data were adjusted for sample dilution and expressed as neutralising antibody ug/ml as determined using the reference standard.

### Activation Induced Marker Assay

Cryopreserved PBMC were thawed and rested for at least 6 hours in filtered R10 - RPMI+10%FBS (Sigma). Cells were counted and divided between two wells of a round bottom 96-well plate (1-3x10^6^/well) in a final volume of 200ul, purified anti-CD40 antibody (Biolegend) was included at a final concentration 1μg/ml. Cells were then stimulated with a pool of overlapping peptides from Wuhan sequence SARS-CoV-2 Spike (JPT technologies) at final concentration of 1μg/ml per peptide, or DMSO as an unstimulated control. Cells were incubated at 37°C overnight for 18 hours. Following stimulation, plates were spun for 2 min at 250g, and 150ul of supernatant removed and immediately frozen at -80°C. Cells were transferred to FACS tubes and washed with cold wash buffer (PBS+0.5% BSA+0.1% EDTA), Fc-block was added for 5 minutes (Biolegend), An antibody mastermix was made using 50μl of brilliant staining buffer (BD biosciences) per sample, to which appropriate volumes of antibody were added as shown in **Supplementary Table 4**. Antibody was added to the cell suspension and cells stained at 4°C for 30 minutes. Cells were then washed and fixed with 1.6% paraformaldehyde for 30 minutes at RT in the dark, washed and run on a BD Symphony A3 flow cytometer (BD Biosciences). Data was collected using BD FACS Diva 8 and analysis was carried out using FlowJo v10.7.1.

### Cytokine Release Profiling

Supernatants from AIM assay cultures were assessed using a LEGENDplex CD8/NK cytokine-profile 13-plex kit (Biolegend) following manufacturer’s instructions. Samples were analysed in duplicate. Cytokine levels were normalised to be equivalent to 1x10^6^ cells per well, cytokine levels in DMSO controls were then removed. Data was analysed using LEGENDplex v8.0 Software (Biolegend).

### IFN-γ ELISpot

T-cell responses were measured using an IFN-γ ELISpot Pro kit (Mabtech) as previously described (15). Briefly, fresh PBMC were rested overnight prior to assay and 0.25-0.3x10^6^ PBMC were added in duplicate per well containing either pep-mix, anti-CD3 (positive) or DMSO (negative) control. Samples were incubated for 16-18hrs. Plates were developed following the manufacturer’s instructions and read using an AID plate reader (AID).

Pepmixes pool containing 15-mer peptides overlapping by 10aa from either SARS-CoV-2 spike S1 or S2 domains from the Wuhan or Omicron (B1.1.529) variant were purchased from JPT technologies.

### Data Visualisation and Statistics

Data was visualised and statistical tests, including normality tests, performed as indicated using GraphPad Prism v9 software. Only results found to be significant (p<0.05) are displayed.

## Data Availability Statement

The raw data supporting the conclusions of this article will be made available by the authors, without undue reservation.

## Ethics Statement

The studies involving human participants were reviewed and approved by PHE Research Ethics and Governance Group (REGG) reference NR0264. Written informed consent to participate in this study was provided by the participants’ legal guardian/next of kin.

## Author Contributions

Conceptualization: AD, GI, PM, and SL. Methodology: AD, CD, and BW. Formal analysis: AD, CD, and BW. Investigation: AD, CD, SS, and NL. Resources: AP, MA, EJ, JGu, PS, ES, SN, GI, IO, BW, JB, SA, JGa, AB, BB, MW, AC, ML, JK, JC, MR, EL, JP, GA, KB, RA, DW, and JW. Data curation: AD and AP. Writing – original draft: AD, AP, PM, and SL. Writing - review and editing: all authors. Visualization: AD, PM, and SL. Supervision: AD, RB, DW, CD, BW, JZ, PM, and SL. Project administration: RB, AP, GI, PM, and SL. Funding acquisition: CD, BW, PM, and SL. All authors contributed to the article and approved the submitted version.

## Funding

This work was partly funded by UKRI/NIHR through the UK Coronavirus Immunology Consortium (UK-CIC) (PM). The study was also funded in part by the MRC (MC UU 1201412) (CD and BW).

## Conflict of Interest 

MR received funding for the COV-BOOST trial under contract *via* University Hospital Southampton NHS Foundation Trust, Funded by the UK NIHR/Vaccine Task Force (NIHR203292). Post-marketing surveillance reports on pneumococcal and meningococcal infection have been provided to vaccine manufacturers for which a cost recovery charge was made to GSK and Pfizer.

The remaining authors declare that the research was conducted in the absence of any commercial or financial relationships that could be construed as a potential conflict of interest.

## Publisher’s Note

All claims expressed in this article are solely those of the authors and do not necessarily represent those of their affiliated organizations, or those of the publisher, the editors and the reviewers. Any product that may be evaluated in this article, or claim that may be made by its manufacturer, is not guaranteed or endorsed by the publisher.
