# Supplementary material for: mRNA or ChAd0x1 COVID-19 Vaccination of Adolescents Induces Robust Antibody and Cellular Responses With Continued Recognition of Omicron Following mRNA-1273
Source: Front Immunol. 2022 Jun 2;13:882515. doi: 10.3389/fimmu.2022.882515 (PMC9201026; doi:10.3389/fimmu.2022.882515)
Supplement: Supplementary file 1 [file DataSheet_1.docx]

**
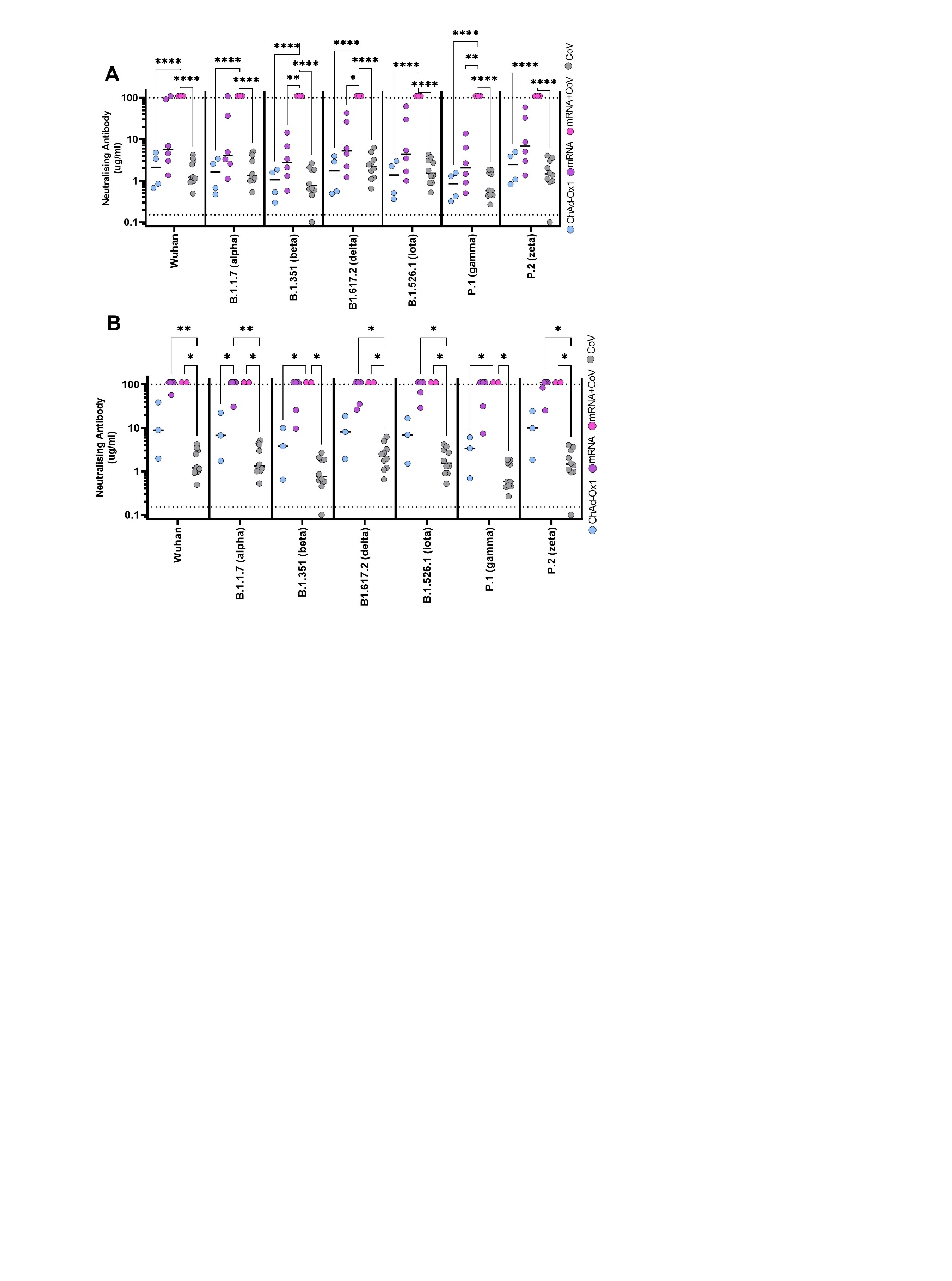
Supplementary Figure 1. Level of antibodies inhibiting binding of spike from viral variants to the ACE2 receptor.** The level of antibody able to inhibit receptor binding by viral variant spike was determined using a Spike-ACE2 receptor binding inhibition MSD assay following first (A) or second (B) dose of vaccine. (A) ChAdOx1 – seronegative children receiving ChAdOx1 vaccination, (n=4), mRNA – seronegative children receiving mRNA vaccination (n=6), CoV+mRNA – seropositive children receiving mRNA vaccination (n=3)). Antibody levels following natural COVID-19 infection (CoV, (n=10)) are shown for comparison. (B) ChAdOx1 – seronegative children receiving ChAdOx1 vaccination, (n=3), mRNA – seronegative children receiving mRNA vaccination (n=5), CoV+mRNA – seropositive children receiving mRNA vaccination (n=2)). Antibody levels following natural COVID-19 infection (CoV, (n=10)) are shown for comparison. Mixed-effect model with Geisser-Greenhouse correction and Tukey multiple comparisons test; * p<0.05, ** p<0.01, *** p<0.001, **** p<0.0001.

**
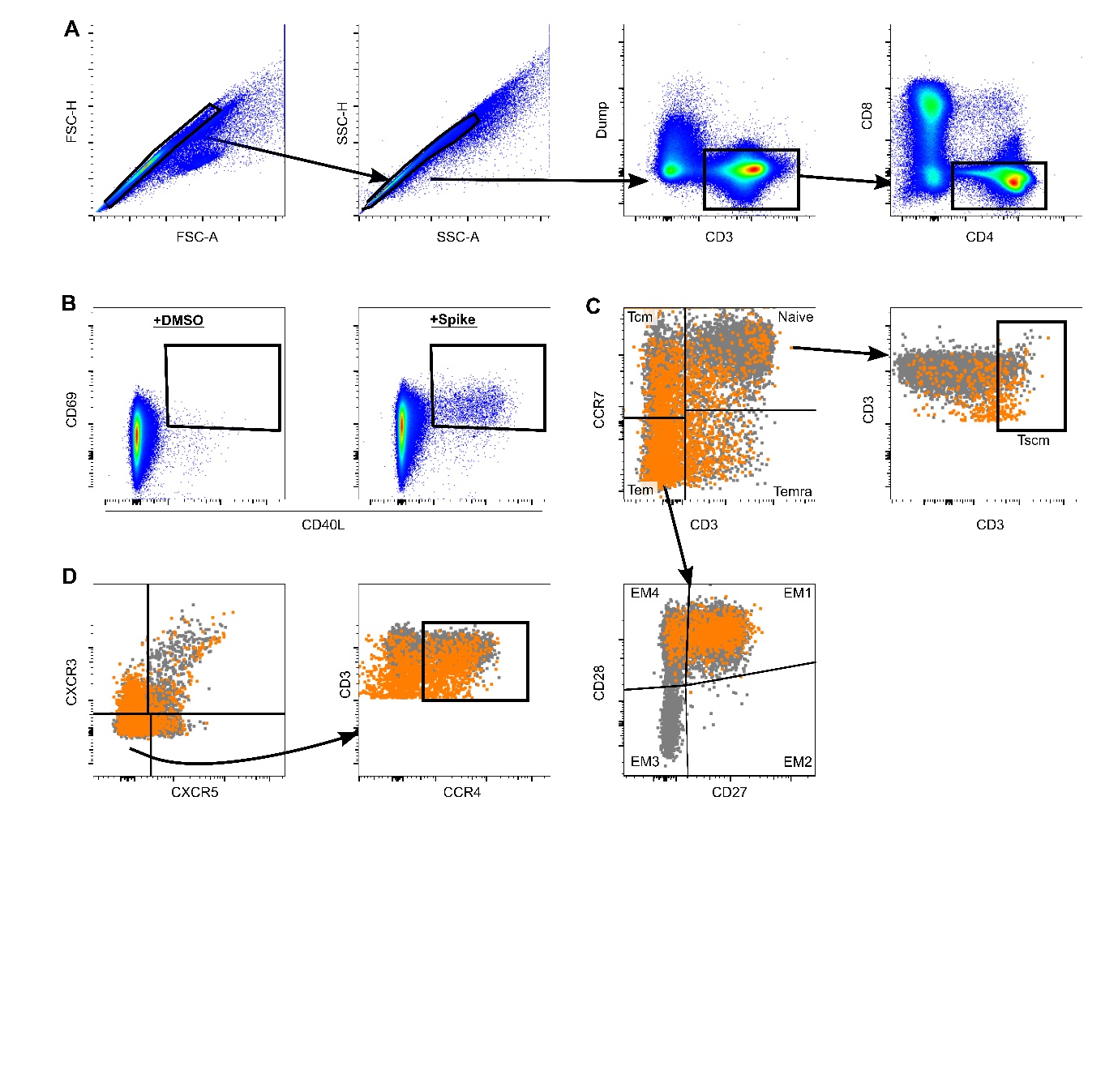
Supplementary Figure 2. Gating strategy for analysis of cellular responses.** A) shows the initial gating strategy to identify CD4 T-cells as shown. B) shows an example of AIM assay staining from a mRNA vaccinated child, showing negative control (+DMSO) and cultures stimulated with overlapping spike peptides. C&D) show examples of further gating and staining from the same donor, the AIM-positive population is shown (orange) and the AIM-negative population (grey). C) Shows assessment of memory populations, Naïve (CD45ra+CCR7+) population were sub-gated to identify Tscm (CD45RA+CCR7+CD95+), likewise the Tem (CD45RA-CCR7-) population was sub-gated and EM1-4 populations defined by CD27/CD28 expression. D) CXCR3 and CXCR5 populations were first identified, and CCR4 expression assessed on the CXCR3-CXCR5- population.

**
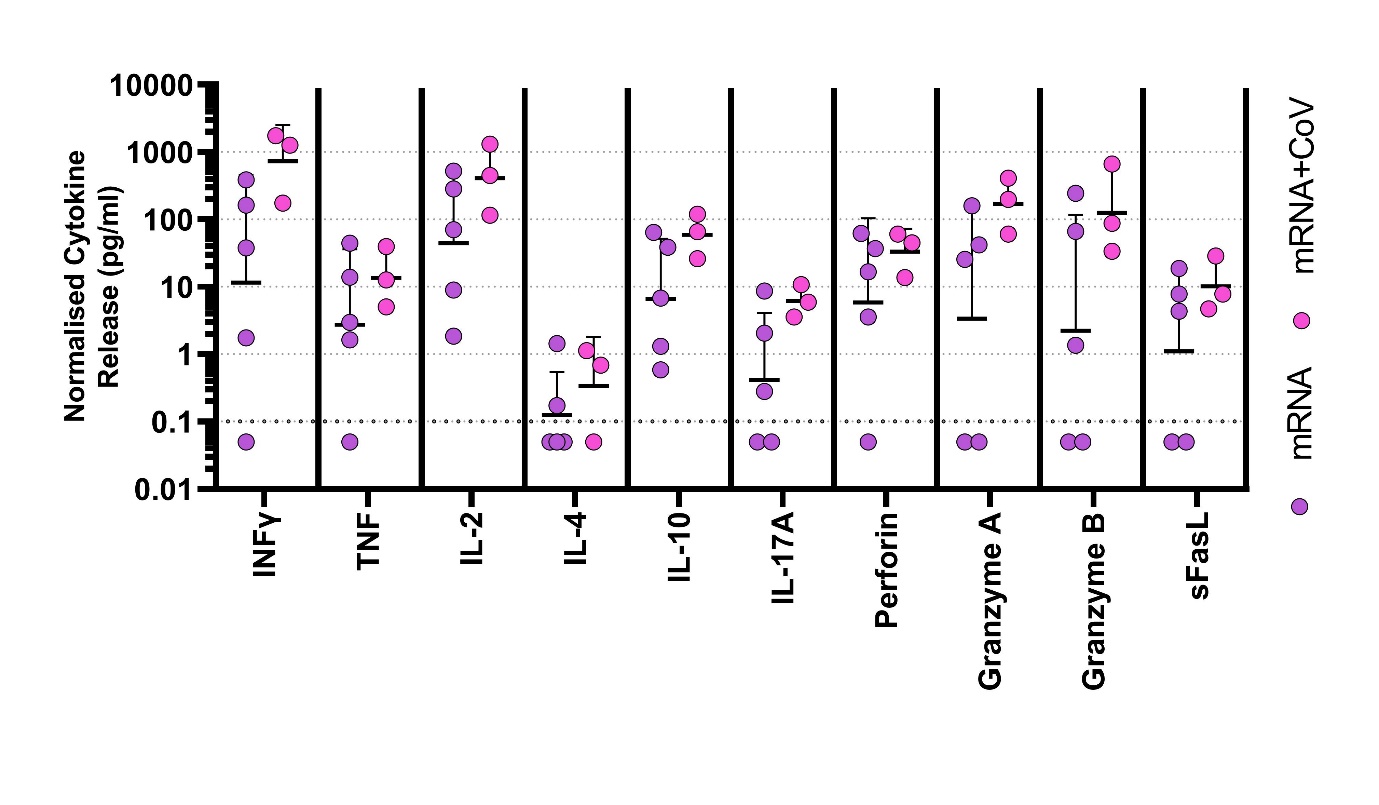
Supplementary Figure 3. Cytokine responses in adolescents following first COVID-19 vaccination.** Supernatant from overnight stimulated cultures of PBMC from mRNA vaccinated adolescents following first vaccine were analysed to identify cytokine production. Data was normalised to 1x10^6^ PBMC per well, cytokine release is shown minus background cytokine production in unstimulated (DMSO) wells. Dots indicate individual donors, bars indicate geometric mean±geo.SD.

**Supplementary Table 1. Demographics and Vaccination details**

| **Cohort** | **ChAdOx1** | **mRNA** | **CoV+mRNA** | **CoV** |
| --- | --- | --- | --- | --- |
| **Donors** | n=6 | n=6 | n=3 | n=10 |
| **Age** | 13 (12-16) | 14 (13-15) | 14 (13-15) | 11 (10-13) |
| **Gender (Male:**  **Female)** | 2:1 | 2:1 | 2:1 | 3:2 |
| **Vaccine** | ChAdOx1 nCoV-19 | mRNA-1273 (n=5),  BNT162b2 (n=1) | mRNA-1273 (n=3) | - |
| **Dose two Interval** | 10.57 weeks  (10.14-10.57) | 11.43 weeks  (9.00-13.14) | 11.43 weeks  (11.43-16.29) | - |
| **Time from PCR positive** | - | - | - | 18.08 weeks  (12.40-21.40) |
| **Comorbidities** | | | | |
| Cerebral palsy | n=2 | n=3 | - | - |
| Congenital muscular dystrophy | n=1 | - | n=1 | - |
| Congenital metabolic disorder | n=1 | - | - | - |
| Down syndrome with neurological complications | n=1 | - | - | - |
| Epilepsy with severe learning difficulties | n=1 | n=5 | n=2 | - |
| Alpha-thalassemia x-linked intellectual disability (ATRX) syndrome | - | n=1 | - | - |

|  | **Vaccine type** | **Spike AU/ml (±SD)** | **RBD AU/ml (±SD)** | **Spike BAU/ml (±SD)** | **RBD BAU/ml (±SD)** |
| --- | --- | --- | --- | --- | --- |
| **Dose 1** | **ChAdOx1** | 10259±2.88 | 4695±3.06 | 92.43± 0.03 | 127.7±0.08 |
|  | **mRNA** | 44856±3.05 | 14497±3.11 | 404.15±0.03 | 394.31± 0.08 |
|  | **CoV+mRNA** | 352762±1.22 | 300969±1.58 | 3178.38±0.01 | 8186.35± 0.04 |
| **Dose 2** | **ChAdOx1** | 36519±2.03 | 19070±2.11 | 329.03±0.02 | 518.7± 0.06 |
|  | **mRNA** | 239519±2.12 | 156116±3.34 | 2158.06±0.02 | 4246.35± 0.09 |
|  | **CoV+mRNA** | 396470±1.01 | 359518±1.04 | 3572.19±0.01 | 9778.89± 0.03 |
| **~3mths** | **ChAdOx1** | 23516±1.83 | 11764±1.93 | 211.88±0.02 | 319.98±0.05 |
|  | **mRNA** | 198840±2.71 | 100354±3.25 | 1791.55±0.02 | 2729.63±0.09 |
|  | **CoV+mRNA** | - | - | - | - |
|  | **CoV** | 11059±1.88 | 4398±1.98 | 99.64±0.02 | 119.63±0.05 |

**Supplementary Table 2. Antibody titres following vaccination.** Shown are Geo. Mean titres for Spike and RBD for each group as indicated following first or second dose, as shown in Figure 1A and 1B. Data are presented as AU/ml, data are also provided as BAU/ml compatible with the WHO reference standard.

|  | **SAFE-KIDS** | | | **Children (4-11 years)*** | | | **Adults*** | | |
| --- | --- | --- | --- | --- | --- | --- | --- | --- | --- |
| Antigen | Geo. mean | Range | n | Geo. mean | Range | n | Geo. mean | Range | n |
| OC43-Spike | 18695 | 890-142727 | 15 | 19296 | 2610  134346 | 48 | 30526 | 4795-133678 | 64 |
| HKU-1-Spike | 2979 | 108-15099 | 15 | 3544 | 57.83  24190 | 48 | 9090 | 415-86245 | 64 |
| NL-63-Spike | 1871 | 176-8874 | 15 | 1899 | 43.75  9157 | 48 | 2643 | 422-13145 | 64 |
| 229e-Spike | 5905 | 12-117530 | 15 | 4845 | 52.4  93068 | 48 | 17032 | 187-244566 | 64 |
|  |  |  |  |  |  |  |  |  |  |
| Influenza A-Michigan H1 | 49409 | 2386  426286 | 15 | 38226 | 33.94  426598 | 91 | 39571 | 2661-1061609 | 154 |
| Influenza A- Hong Kong H3 | 60923 | 1357  437984 | 15 | 81171 | 411.2  897645 | 91 | 38078 | 275-937912 | 154 |
| Influenza A- Shanghai H7 | 310.3 | 31.12  2898 | 15 | 773 | 32.56-  12000 | 91 | 3768 | 33-57602 | 154 |
| Influenza B-Brisbane HA | 20380 | 1384  100580 | 15 | 15897 | 185.2  129044 | 91 | 46108 | 1434-847502 | 154 |
| Influenza B-Phuket HA | 14257 | 2604  134598 | 15 | 18782 | 216.4  241222 | 91 | 55756 | 1426-866480 | 154 |
| RSV-Pre Fusion F | 139004 | 49578  562442 | 15 | 114251 | 23768  454518 | 91 | 146751 | 1136-849109 | 154 |

**Supplementary Table 3. Antibody titres to other respiratory viruses.** Antibody levels to the indicated antigens were determined using MSD. Results are shown as the geometric mean and range of each cohort as shown. Coronavirus antibody titres are shown for baseline samples from the SAFE-KIDS (vaccine) study, or seronegative children and adults. Influenza and RSV (Respiratory syncytial virus) antibody titres are the average of timepoint tested. *Antibody titres from a previously published data set (15)

| Antigen | Fluorochrome | Supplier | Final Dilution |
| --- | --- | --- | --- |
| CD3 | BUV805 | BD Bioscience | 1/100 |
| CD4 | BV750 | Biolegend | 1/100 |
| CD8 | BV510 | Biolegend | 1/100 |
| CD45RA | BUV395 | BD Bioscience | 1/200 |
| CCR7 | APC-Fire-750 | Biolegend | 1/20 |
| CD27 | Fitc | Biolegend | 1/50 |
| CD28 | BV650 | Biolegend | 1/20 |
| CD95 | AF700 | Biolegend | 1/50 |
| CXCR5 | PE-Cy7 | Biolegend | 1/25 |
| CXCR3 | APC | Biolegend | 1/25 |
| CCR4 | BV605 | Biolegend | 1/50 |
| CD69 | BV786 | Biolegend | 1/25 |
| CD154 (CD40L) | PE-Dazzle-594 | Biolegend | 1/25 |
| CD137 (4-1BB) | PE | Biolegend | 1/25 |
| CD14/CD19 | BV570 | Biolegend | 1/100 |
| Fixable Viability stain | FVS570 | BD Bioscience | 1/1000 |

**Supplementary Table 4. Flow Cytometry antibody suppliers and dilutions**
